# 1055. Comparison of Scoring systems in Predicting Clinical Outcomes in Patients Hospitalized with COVID 19

**DOI:** 10.1093/ofid/ofac492.896

**Published:** 2022-12-15

**Authors:** Anderson Huang, Kevin Dieckhaus, Lisa M Chirch, Roopjeet K Bath, Jessica Abrantes-Figueiredo, Jennifer Onwochei, Assaf Holtzman, Neelam Tailor, Chia-Ling Kuo

**Affiliations:** UCONN - - Farmington, CT, Glastonbury, Connecticut; UConn Health, Southington, Connecticut; University of Connecticut School of Medicine, Farmington, CT; UConn Health, Southington, Connecticut; Saint Francis Hospital, Hartford, Connecticut; University of Connecticut, Hartford, Connecticut; University of Connecticut School of Medicine, Farmington, CT; University of Connecticut Health, West Hartford, Connecticut; University of Connecticut Health, West Hartford, Connecticut

## Abstract

**Background:**

Previous scoring systems have been proposed to predict COVID19 outcomes, however none have been universally adopted. Two scoring systems of interest are Monoclonal Antibody Screening Score (MASS) and Oral Antiviral and Monoclonal Antibody Screening Score (OMASS). MASS prioritized patients for outpatient monoclonal antibody treatment based on risk of hospitalization, and OMASS was a modified version of MASS used to prioritize outpatient oral antivirals. We created a modified scoring system (UCH2021) incorporating vaccination status. These scores (table 1) have not been used to predict in-hospital clinical outcomes. We investigate these systems’ abilities to predict mortality and oxygen requirements in hospitalized COVID19 patients. They do not require blood tests and allow for more rapid triage.

**Table 1:**
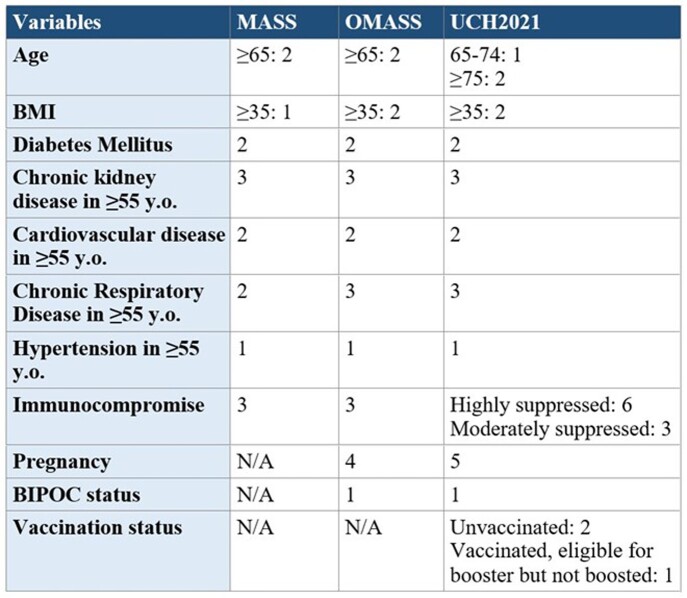
MASS, OMASS, UCH2021 Scoring Criteria

**Methods:**

A retrospective chart review was performed on 133 patients in two tertiary care centers between March and Sept. 2020 with RT-PCR confirmed SARS CoV2. Baseline risk factors were collected and MASS, OMASS, and UCH2021 were calculated. Primary outcomes included mortality, need for intubation, and need for supplemental oxygen >6L during hospitalization. Secondary analysis assessed if any individual risk factors were associated with those outcomes. These systems were evaluated via area under the curve calculations. Two groups based on an outcome were compared using two-sample t-tests for continuous variables and Fisher’s exact tests for categorical variables.

**Results:**

All three systems demonstrated some discriminative power for mortality (table 2), but not for oxygen and intubation requirements. There was statistically significant difference in age between survivors and deceased (table 3), and BMI for oxygen requirements (table 4). Other risk factors were not predictive of mortality or oxygen requirement.

**Table 2:**
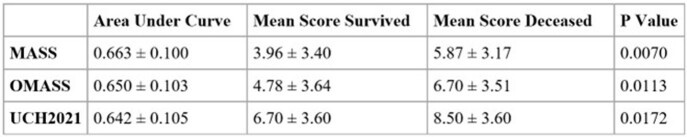
MASS, OMASS, UCH2021 Scores and Mortality in Hospitalized COVID19 Patients

**Table 3:**
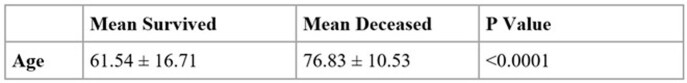
Age and Mortality in Hospitalized COVID19 Patients

**Table 4:**
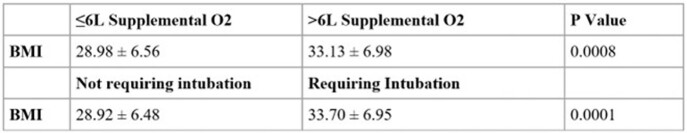
BMI and Oxygen Requirements in Hospitalized COVID19 Patients

**Conclusion:**

The MASS, OMASS, and UCH2021 score all had predictive power in determining in-hospital mortality, with moderate accuracy, however none were predictive of oxygen requirements. Age and BMI were also good predictors of mortality and oxygen requirements respectively. This study was completed prior to vaccine distribution in the US. Further studies would be helpful to assess if UCH2021 score has greater discriminative power in samples with vaccinated patients.

**Disclosures:**

**All Authors**: No reported disclosures.

